# Cadmium-Induced Kidney Apoptosis Based on the IRE1α-XBP1 Signaling Pathway and the Protective Effect of Quercetin

**DOI:** 10.3390/toxics13020129

**Published:** 2025-02-10

**Authors:** Liuxin Wang, Weiwei Cao, Ting Wu

**Affiliations:** 1 College of Food Science and Technology, Huazhong Agricultural University, Wuhan 430070, China; wangliuxin2005@163.com; 2 College of Food and Bioengineering, Henan University of Science and Technology, Luoyang 471023, China; caoweiwei@haust.edu.cn

**Keywords:** cadmium, quercetin, kidney, IRE1α-XBP1, apoptosis

## Abstract

Cadmium (Cd) is an important environmental pollutant that can enter the body and inflict kidney damage. Quercetin (Que) is a natural flavonoid compound that can alleviate kidney damage in Cd-treated rats, but the specific mechanism is unclear. Herein, 24 male Sprague–Dawley rats were divided into four groups, namely the control, Cd, Cd + Que, and Que groups. Four weeks later, the rats were anesthetized with ether and were euthanized; then, their blood was collected and their kidneys were removed. Renal function markers were measured. Kidney tissue structure was observed by HE staining, cell apoptosis was detected by the TUNEL method, and mRNA and protein expression levels in the IRE1α-XBP1 apoptosis signaling pathway were analyzed by RT-PCR and Western blotting. Results showed that the Cd treatment group exhibited decreased renal dysfunction and pathologic injury. Cd-induced tissue damage and cell apoptosis and significantly increased the mRNA and protein expression levels (*p* < 0.01) related to the IRE1α-XBP1 signaling pathway. Compared with the Cd group, the Cd + Que group exhibited increased renal dysfunction. Conversely, kidney tissue damage and renal cell apoptosis decreased, and the mRNA and protein expression levels of IRE1α and XBP1 significantly decreased (*p* < 0.01). Cd treatment inflicted renal damage. Therefore, Que can restore the kidney tissue damage and alleviate the cell apoptosis caused by Cd through the inhibition of the IRE1α-XBP1 signaling pathway.

## 1. Introduction

Cadmium (Cd) is a highly toxic heavy metal that exists in the natural environment. It cannot be biodegraded and is prone to long-term accumulation in the body, thereby damaging tissues and organs [1]. Despite various measures taken by many countries to control Cd pollution, it still exists in many regions. Cd has wide-ranging applications in industry and agriculture, such as printing and dyeing, electroplating, insecticides, fungicides, paints, and other industries. The sources of Cd pollution in the environment are very complex. The first type is natural sources, such as rock weathering caused by natural activities, and the second type is anthropogenic sources. For example, Cd primarily originates from industrial production (e.g., the smelting of copper, lead, and zinc), the production of aviation materials, and the manufacturing of electrical appliances. It may also come from the discharge of Cd-containing wastewater, the widespread use of Cd containing fertilizers and pesticides in agricultural production, and the non-professional disposal of cadmium batteries. Cd in the environment enters human or animal bodies through biological enrichment, including the food chain, leading to the occurrence of Cd-poisoning-related diseases [2].

Cd accumulation in the body is caused by inhalation, ingestion, and even direct contact with contaminated air, water, and food. Cd is transported into various parts of the body after entering it, easily accumulating in multiple organs. Cd is difficult to eliminate for a long time due to its long half-life, leading to diseases in tissues or organs such as the kidneys, blood, liver, and testes. Renal injury is usually caused by chronic exposure to Cd, and the condition is difficult to reverse or may even be irreversible.

The kidneys are the main metabolic and target organs of Cd poisoning [3]. When Cd poisoning occurs, the Cd metallothionein complex in tissue cells enters the bloodstream due to the filtering effect of the glomerulus. It further reaches the kidneys, where it is reabsorbed by the renal tubules and continuously accumulates after chronic Cd exposure [4]. Long-term exposure to Cd can lead to metabolic disorders and the excessive accumulation of Cd in the kidneys, as well as calcium loss in the body. Cd damages the kidneys, manifesting as changes in the renal function indicators: uric acid (UA), creatinine (Cre), or blood urea nitrogen (BUN). It can even induce renal cell death [5,6]. The mechanism of action of Cd primarily involves activating the endoplasmic reticulum stress (ERS) pathway, leading to cell apoptosis in chicken kidney [7]. Fish treated with CdCl_2_ experienced oxidative stress that altered Nrf2-Keap1 signaling and triggered piscine head kidney macrophage apoptosis [8].

The endoplasmic reticulum (ER) is the core of the eukaryotic cell membrane system, accounting for approximately 50% of the cell membrane. According to whether ribosomes are attached to the lumen surface, ER can be divided into the rough endoplasmic reticulum or smooth endoplasmic reticulum. The rough ER has a large number of ribosomes attached to its surface, presenting a granular appearance and mostly large flat membrane vesicles arranged neatly. The surface of the smooth ER is relatively smooth, without ribosome attachment, and is usually small and tubular or vesicular [9]. Excessive accumulation of nonfolding proteins in the ER can cause an imbalance in the ER environment, leading to ERS. Short-term or mild ERS can enhance protein folding ability, promote the degradation of abnormal proteins, and re-establish the balance of the ER environment. However, strong or sustained ERS can lead to an unfolded protein response (UPR), thereby promoting the activation of caspase-12-dependent apoptotic pathways in cells, leading to cell apoptosis and even cell death [10,11]. UPR activation is mediated by three stress sensors: inositol dependent enzyme 1 alpha (IRE1α), transmembrane receptor protein kinase RNA-like ER kinase (PERK), and activated transcription factor 6. These stress sensors are combined with ER partner GRP78 under nonstress conditions. ERS such as stress caused by the accumulation of unfolded proteins activates UPR by separating the stress sensor from GRP78 [12].

Apoptosis is the autonomous and orderly death of cells controlled by genes. UPR can lead to cell apoptosis caused by ER overload when ERS is excessive and steady-state reconstruction fails. ERS activates apoptosis as follows [13,14]. (1) In the IRE1α/XBP1 pathway, activated IRE1α-activating enzyme activity further cleaves the mRNA of XBP to produce transcription factors encoding XBP1, causing a large accumulation of unfolded proteins in the cell. These abnormally accumulated proteins activate the UPR through the IRE1α/XBP1 pathway and induce cell apoptosis. (2) In the PERK pathway, PERK undergoes biochemical processes such as autophosphorylation, leading to the phosphorylation of eIF2 α and expression of ATF4. (3) The ATF6 apoptosis pathway involves the dissociation of ATF6 from GRP78 protein and its transfer to the Golgi apparatus. It is cleaved by S1P and S2P to form cleaved ATF6, which enters the nucleus and activates CHOP gene expression, inducing cell apoptosis.

Quercetin (Que) is a flavonol compound with multiple biological activities [15]. It exists in high abundance in the leaves, fruits or seeds of many plants, with higher levels found in onions, kale, grapes, wine, green tea, and apples. Que has excellent biological and pharmacological activities, including antioxidant effects, the inhibition of an angiotensin-converting enzyme, anti-inflammatory effects, antibacterial effects, anticancer properties, and antiviral effects [16]. Que exists in the form of glycosides and has high medicinal value, making it very beneficial to health. It can also be used as a food supplement. However, due to its short half-life, low water solubility, and rapid metabolism in the body, its bioavailability is relatively low [17,18].

In the present research, SD rats were treated with Cd and/or Que. The action of the IRE1α-XBP1 signaling pathway in Cd-induced nephrotoxicity was investigated, to elucidate its association with Cd-induced renal apoptosis under ERS and the protective effect of Que.

## 2. Materials and Methods

### 2.1. Chemicals and Reagents

Sprague–Dawley (SD) rats were bought from Henan Experimental Animal Center (Zhengzhou, China). CdCl_2_ (99.95%) was purchased from Aladdin (Shanghai, China), and Que (97%) was provided by Rhawn (Shanghai, China). qPCR regents and TRIzol were purchased from Vazyme (Nanjing, China). Serum uric acid (UA), creatinine (Cre), and blood urea nitrogen (BUN) kits were bought from Nanjing Jiancheng Bioengineering Institute (Nanjing, China). Anti-GRP78 (Boster Biological Technology Co., Ltd., Pleasanton, CA, USA), Anti-IRE1α, anti-XBP1, anti-Caspase-12, and anti-Bcl-2 primary antibodies (Proteintech Biotechnology, Rosemont, IL, USA) were also used. Anti-β-actin and anti-Caspase-3 primary antibodies we obtained from ABclonal Technology, Woburn, MA, USA.

### 2.2. Animal Care and Sample Treatment

Twenty-four male SD rats were housed in an animal house (temperature: 20 °C, humidity: 50%) for 1 week and then grouped randomly. The following groups were established: a control group that received 0.9% NaCl injected intraperitoneally, a Cd treatment group that received 2 mg/kg b.w. CdCl_2_, a Cd + Que co-treatment group that received 2 mg/kg b.w. CdCl_2_ and 100 mg/kg b.w. Que, and a Que-treatment group that received 100 mg/kg b.w. Que. CdCl_2_ was injected intraperitoneally every day, and Que was administered through oral gavage every day. The experiment lasted for 28 days. The rats were anesthetized with diethyl ether, blood was collected from the veins to evaluate kidney function, and kidneys were removed. One kidney of each rat was fixed in 4% paraformaldehyde solution for 24 h to conduct tissue slicing. The other kidney was kept at −80 °C for RT-PCR and Western blot analysis.

### 2.3. Evaluation of Kidney Function

The obtained blood was stored until it coagulated and centrifuged for 12 min to collect the serum at 2500 rpm. UA and Cre levels were measured using an enzyme labeler (Tecan, Männedorf, Switzerland). Serum BUN concentrations were measured with a spectrophotometer (MAPADA, Shanghai, China).

### 2.4. Hematoxylin and Eosin (HE) Staining Analysis

Kidneys were fixed with a 4% paraformaldehyde solution for at least 24 h. Then, the tissue was cut into 0.5 cm × 0.5 cm × 0.5 cm blocks and embedded. A paraffin microtome was used to slice the tissue and bake it. The slices were placed in xylene and ethanol sequentially for dewaxing followed by HE staining. The stained slices were placed in ethanol of different concentrations for dehydration, made transparent with xylene, and sealed with neutral gum. Pathological changes in kidney tissue were observed with a microscope.

### 2.5. TUNEL Method

Paraffin sections were deparaffinized with xylene, soaked in gradient ethanol for dehydration, and then washed three times with PBS. After reacting with the added DNase-free protease at 37 °C for 15 min, the slices were washed three times with PBS. After adding 50 μL of the TUNEL detection solution, the samples were incubated in the dark for 1 h. Nuclei were stained with DAPI, the anti-fluorescein quencher was sealed, and photos were taken under a fluorescence microscope (3D histech, Budapest, Hungary).

### 2.6. RT-PCR

The kidney sample was ground in TRIzol lysis buffer to extract RNA. Using gDNA Wiper Mix for the reverse transcription synthesis of cDNA, and then using cDNA as a template, fluorescence quantitative PCR was performed using HiScript III qRT SuperMix. The primer sequences for the target genes are listed in Table 1.

### 2.7. Western Blot Analysis

About 50 mg of renal cortex tissue was collected and placed in a 1.5 mL centrifuge tube. RIPA lysis buffer containing a mixture of phosphatase inhibitor and protease inhibitor were added, and the sample was ground thoroughly on ice. The sample was centrifuged, and the supernatant was collected. The total protein concentration was measured, and the protein was denatured. Amounts equal to 50 μg of a protein sample from each group were placed on 12% sodium dodecyl sulfate polyacrylamide gel for electrophoresis, and the gels were transferred onto PVDF membranes for 2 h. Then, 5% milk was prepared using TBS with Tween-20 added, and the membranes were blocked at 37 °C for 2 h. After washing with TBST, the primary antibody diluted with a dilution buffer was incubated overnight on the membrane at 4 °C. The primary antibodies used were as follows: the anti-GRP78, anti-IRE1α, anti-XBP1, anti-Caspase-12, anti-Bcl-2, anti-Caspase-3, and anti-β-actin primary antibodies [19]. Membranes underwent TBST washing, followed by incubation with goat anti-rabbit IgG goat polyclonal antibody (1:10,000) or anti-mouse IgG goat polyclonal antibody (1:10,000) for 1 h at 37 °C. Specific protein bands were observed using ECL chemiluminescence kits and a Chemiluminescence gel imager (Omega LumC, Aplegen, San Francisco, CA, USA). Finally, Image J software (version: 1.51j8) was used to analyze the related protein gray value, which was normalized to the *β*-actin protein level.

### 2.8. Statistical Analysis

SPSS version 27.0 was used for data analysis. Data were analyzed by one-way ANOVA and the least significant difference (LSD) test, and are presented as the mean ± SD. *p* > 0.05 indicated that the difference was not significant, whereas *p* < 0.05 and *p* < 0.01 indicated significant differences.

## 3. Results

### 3.1. Effects of Que on Renal Function in Cd-Exposed Rats

**Figure 1:**
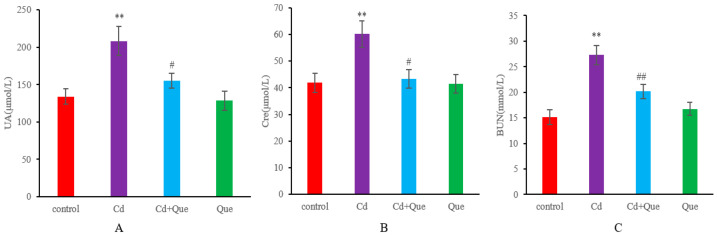
Renal function in Cd-induced kidney injury and the protective effects of Que on serum UA (**A**), Cre (**B**), and BUN (**C**) levels in rats. Data are expressed as the mean ± SD, n = 6. ** *p* < 0.01, compared with the control group. ^#^ *p* < 0.05 and ^##^ *p* < 0.01, compared with the Cd group. The same apply to the figures below.

UA, Cre, and BUN in the serum were detected to investigate the effect of Que on renal dysfunction induced by Cd in rats. As shown in Figure 1A–C, the contents of UA, Cre, and BUN significantly increased after CdCl_2_ injection (*p* < 0.01). With Que treatment, UA, Cre, and BUN levels were considerably lower in the Cd + Que group (*p* < 0.05 or *p* < 0.01), indicating that Que alleviated the renal dysfunction caused by Cd.

### 3.2. Histopathological Injury

**Figure 2:**
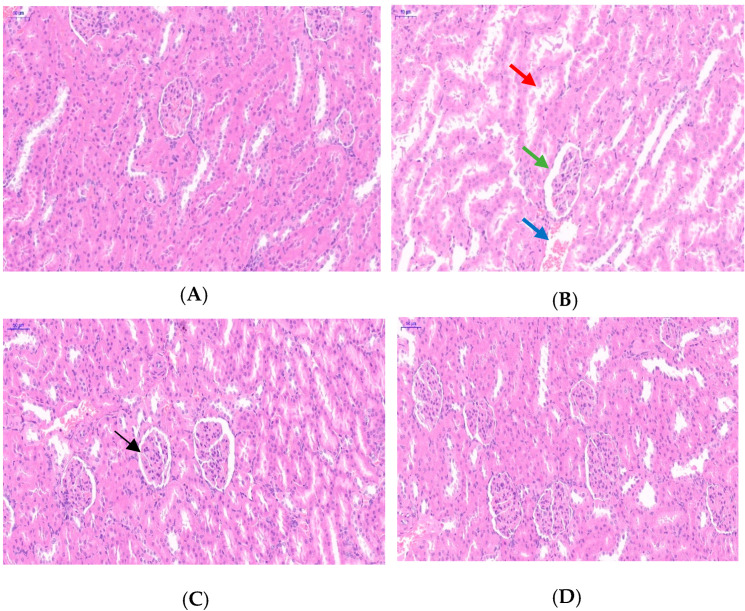
Que reversed the histopathologic changes in Cd-induced renal injury in rats. (**A**) Control, (**B**) CdCl_2_, (**C**) CdCl_2_ + Que, and (**D**) Que. (200×). Scale bars: 50 μm. The red arrow shows the widened renal tubular lumen, and the green arrow shows the reduced glomerular volume and widened glomerular capsule. The blue arrow shows the separation of renal tubular epithelial cells, partial necrosis, and nuclear lysis. The black arrow shows the improved glomerular capsule.

Figure 2 showed that the morphology of renal tubules, glomeruli, and their cystic cavities in the control group’s renal tissue was basically normal. The Cd treatment group showed significantly reduced glomerular volume, widened glomerular capsules, detached and degenerated/necrotic renal tubular epithelial cells with nuclear lysis, and significantly widened lumens. However, the glomerular and tubular changes in the Que treatment group were not significant compared with those of the control group. The degeneration, partial necrosis, and nuclear dissolution of renal tubular epithelial cells were significantly improved in the Cd + Que treatment group. The degree of reduction in glomerular volume and enlargement of the renal tubular lumen was significantly lower in the Cd treatment group.

### 3.3. TUNEL Observation of Renal Cell Apoptosis

**Figure 3:**
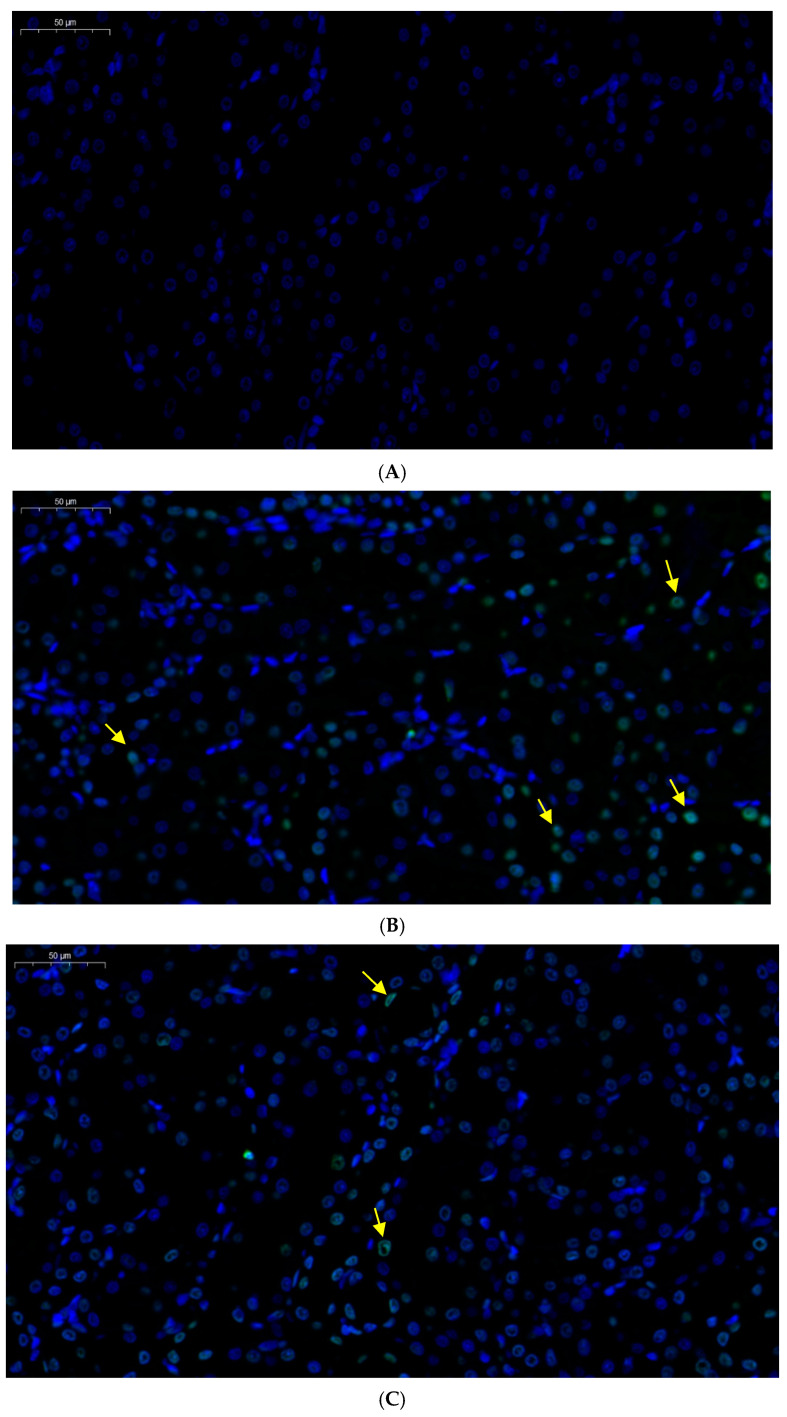

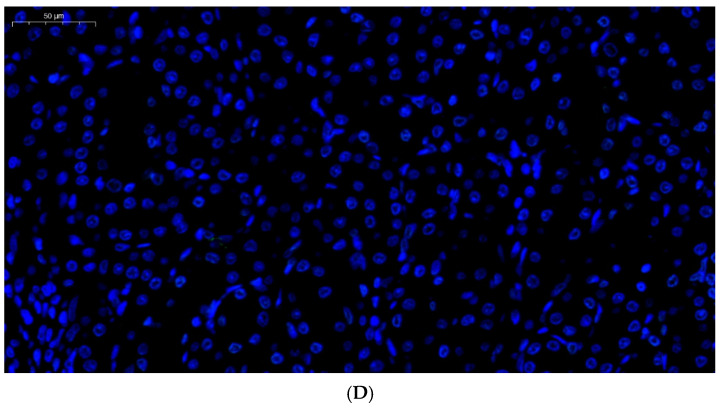
Que, administered by the TUNEL method, reversed the apoptosis of cells in rats with Cd-induced renal injury. (**A**) Control, (**B**) CdCl_2_, (**C**) CdCl_2_ + Que, and (**D**) Que. Scale bars: 50 μm. The yellow arrow shows the apoptotic cells.

As shown in Figure 3, the green arrow shows the apoptotic cells. Compared with the control group, no significant differences were observed in the number of apoptotic renal cells in the Que treatment group, although the number of apoptotic cells in the renal tissue of the Cd group increased significantly. However, the number of apoptotic cells in the Cd + Que co-treatment group significantly decreased.

### 3.4. mRNA Expression and Protein Levels of Apoptosis-Related Genes

**Figure 4:**
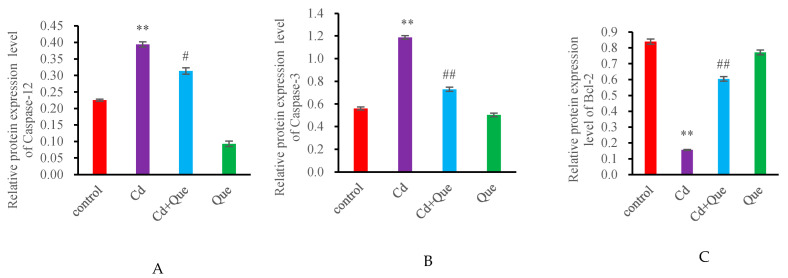
Effects of CdCl_2_ and Que on the expression levels of apoptosis-related genes. (**A**) Caspase-12, (**B**) Caspase-3, and (**C**) Bcl-2. ** *p* < 0.01, compared with the control group. ^#^ *p* < 0.05 and ^##^ *p* < 0.01, compared with the Cd group.

**Figure 5:**
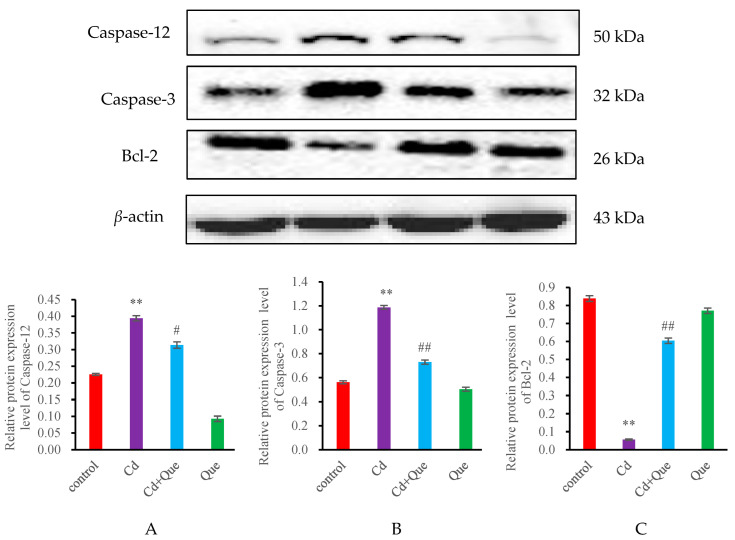
Effects of CdCl_2_ and Que on apoptosis-related proteins. (**A**) Caspase-12 and (**B**) Caspase-3 (**C**) Bcl-2. ** *p* < 0.01, compared with the control group. ^#^ *p* < 0.05 and ^##^ *p* < 0.01, compared with the Cd group.

No significant difference was found in the mRNA and protein levels of Caspase-12, Caspase-3 and Bcl-2 in the Que group compared with the control group (Figure 4 and Figure 5). However, the contents of Caspase-12 and Caspase-3 in the Cd-treatment group increased (*p* < 0.01), however the levels of Bcl-2 decreased significantly (*p* < 0.01). Compared with the Cd-treatment group, the mRNA and protein levels of Caspase-12 and Caspase-3 in the Cd + Que-treated group significantly decreased, but Bcl-2 level increased significantly (*p* < 0.01).

### 3.5. mRNA and Protein Levels of ERS-Related Genes

**Figure 6:**
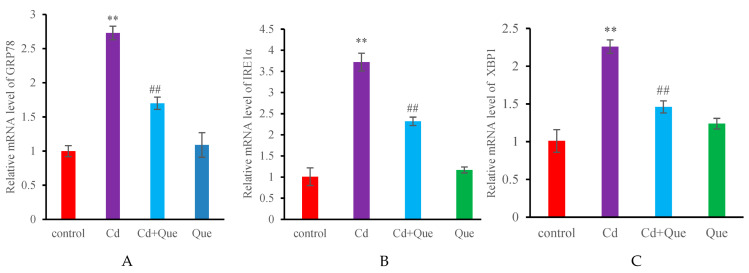
Effects of CdCl_2_ and Que on the expression levels of ER stress-related genes. (**A**) GRP78, (**B**) IRE1α and (**C**) XBP1. ** *p* < 0.01, compared with the control group. ^##^ *p* < 0.01, compared with the Cd group.

**Figure 7:**
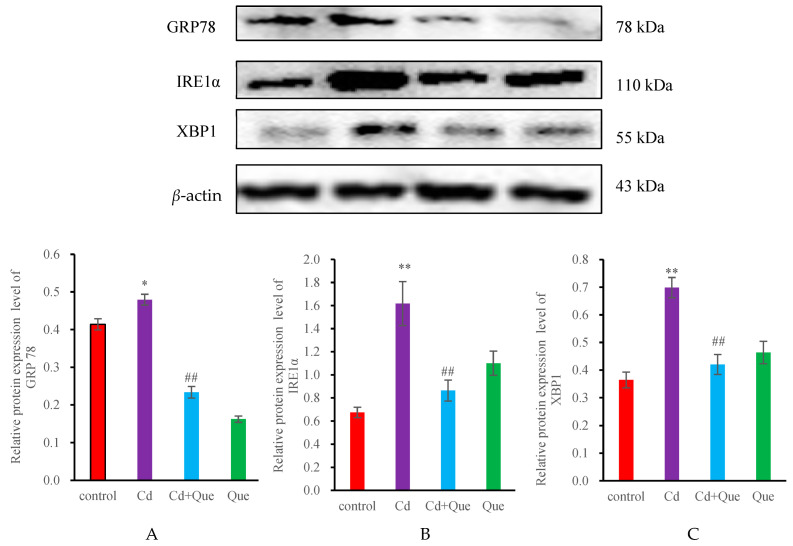
Effects of CdCl_2_ and Que on ER stress-related proteins. (**A**) GRP78, (**B**) IRE1α and (**C**) XBP1. * *p* < 0.05 and ** *p* < 0.01, compared with the control group. ^##^ *p* < 0.01, compared with the Cd group.

Figure 6 and Figure 7 show that, compared with those in the control group, the mRNA and protein levels of GRP78, IRE1α and XBP1 in the Cd-treatment group increased significantly (*p* < 0.01). However, the GRP78, IRE1α, and XBP1 levels in the Cd + Que-treated group significantly decreased compared with those in the Cd-treatment group (*p* < 0.01). No significant differences were observed between the GRP78, IRE1α, and XBP1 levels in the Que group and those in the control group.

## 4. Discussion

Cadmium is an important environmental pollutant. With its increasing use, and with the discharge of wastewater, exhaust gas, and waste residue from industrial electroplating, mining, smelting, dyes, batteries, and the chemical industries [20,21], pollution has become increasingly severe, posing a significant threat to health and environmental hygiene. The kidney is a target organ for Cd poisoning [3]. And cadmium exposure aggravates renal injury by inhibiting autophagy in rats with diabetes [22]. Que is a natural antioxidant substance with biological activities such as antioxidant, antiviral, antihypertensive, and immune regulation activities [16,23,24]. Quercetin is one of the most abundant natural polyphenols in food. Natural quercetin is found in foods such as wine, black tea, and green tea. The recommended daily dose of quercetin in dietary supplements is usually below 1000 mg. Administration of 1000 mg of quercetin and 1000 mg of vitamin C daily is recommended for the treatment of patients who have had one or more chronic diseases for 12 weeks. After treatment with Que, no negative changes were observed in safety parameters such as hematocrit and hemoglobin [18]. In our study, the dose of Que used was 100 mg/kg body weight. Recent studies have shown that Que can effectively alleviate kidney damage caused by Cd [25]. However, the mechanism of Cd-induced kidney injury remains unclear. Whether Que can inhibit Cd-induced kidney injury also warrants further research.

UA is the final product of purine metabolism in the body and is primarily excreted through the kidneys. UA plays an important role in regulating animal growth and reproduction. Under normal circumstances, the production and excretion of UA in the body are in a balanced state. However, when the body produces too much UA and cannot excrete it in a timely manner or its excretion mechanism gradually deteriorates, excessive accumulation of UA can occur. Urea, as the main end-product of protein metabolism in the body, is the main component of non-protein nitrogen in the blood. The process of protein synthesis requires a large amount of amino acids and various non-essential amino acids, with urea being the main one. Due to the effects of kidney failure and nephritis, the content of urea nitrogen in the blood significantly increases, and it is primarily excreted through the kidneys. Cre is a substance produced during the process of muscle energy production, and it is metabolized by phosphocreatine. Healthy kidneys can filter creatinine from the blood. It is a commonly used indicator with which to measure renal filtration function. Therefore, the Cre level in the blood reflects the filtering function of the kidneys and is an important indicator for detecting kidney function. In clinical practice, changes in UA, Cre, and BUN levels are commonly used to determine the conditions of kidney function. When the kidney is damaged, the cellular structure of the kidney is damaged to a certain extent, and the levels of UA, Cre, and BUN increase. This study found that the levels of UA, Cre, and BUN in the serum of Cd-treated rats significantly increased. Huang et al. treated C57BL/6 mice with 2 mg/kg/d CdCl_2_ intraperitoneally, and in agreement with our results, found that the levels of serum urea nitrogen and Cre in the Cd-treated group were higher than those in the control group [26]. Compared with the Cd group, the levels of UA, Cre, and BUN in the Cd + Que-treatment groups significantly decreased. Lead reportedly induces renal oxidative stress, as manifested by significantly increased levels of renal markers such as UA and Cre. Conversely, naringin can significantly attenuate the biochemical changes induced by lead in serum and renal tissue [27]. Rats were orally administered CdCl_2_, and after four weeks, serum levels of UA, Cre, and BUN increased, whereas the Cre clearance rate significantly decreased [28]. Que treatment significantly alleviated the Cd-induced biochemical changes in serum, urine, and renal function. The above results are similar to those of the present experiment, indicating that Cd can cause renal dysfunction in rats, and Que exerted a certain alleviating effect on Cd-induced kidney injury in rats.

The degree of Cd-induced kidney tissue damage is related to factors such as dosage, route, and time of administration [29]. Our results showed that the control and Que groups had renal tubules with a normal structure, glomeruli, and their cystic cavities. However, compared with the control group, the Cd-treated rat kidney showed significantly more significant glomerular atrophy, wider renal glomeruli, and larger tubular volumes (Figure 2). Unlike the Cd treatment group, the number and degree of glomerular atrophies were significantly reduced in the Cd + Que-treatment group, indicating that Que is able to reduce the kidney damage induced by Cd due to its inhibition of Cd toxicity.

Apoptosis is an important pathway of Cd-induced nephrotoxicity injury, and Cd-induced apoptosis can be detected by the TUNEL method [30]. The green fluorescence shows the apoptosis cells. The number of apoptotic cells increased significantly in the Cd-treatment rats. These results indicate that Cd led to renal cell apoptosis, resulting in an increased number of apoptotic cells, consistent with the findings of Ding’s studies, in which they observed cadmium-induced BRL-3A cell apoptosis [19]. However, the number of apoptotic cells in the Cd + Que treatment group was significantly reduced compared with that in the Cd treatment group. Caspase-12 containing cysteine is a unique hydrolytic protease that is specifically bound to the ER membrane and can serve as a marker for the ERS-induced apoptosis pathway. After ERS activation, it can cleave activated Caspase-9, thereby activating Caspase-3, which can decompose cell structure, leading to cell apoptosis. The apoptosis mediated by ERS primarily occurs through Caspase-12 activation [10]. It is well known that ER-resident caspase-12 triggers apoptotic cell death. Mice lacking caspase-12 exhibit resistance to endoplasmic reticulum stress-induced cell apoptosis [31]. Apoptosis has important biological significance and is a phenomenon of autonomous and orderly cell death controlled by genes. It plays an important role in maintaining the stability of the internal environment of the body. Apoptosis is a process strictly controlled by multiple genes, among which Bax is a key regulatory gene. The Bax protein belongs to the Bcl-2 family and is a pro-apoptotic protein [32]. The results of this study showed that compared with the control group, the mRNA and protein expression levels of Caspase-12, Caspase-3, and Bax in the kidneys were significantly increased after Cd exposure. Marta Biagioli et al. obtained similar results. They observed that cadmium decreased ER Ca^2+^ signals. They concluded that Cd-induced NIH 3T3 cell death triggered by ER stress and involving caspase-12 activity could be mitigated [33]. However, the mRNA and protein levels of Caspase-12, Caspase-3, and Bax of the Cd + Que treatment group were significantly reduced. This study indicated that Que is able to reduce Cd-induced renal cell apoptosis.

GRP78 is a marker molecule for ERS. It has multiple physiological functions and is primarily involved in protein folding, the regulation of the ERS response, and tumor occurrence and development, as well as in the regulation of cell migration and the capacity for invasion [34]. The IRE1α/XBP1 pathway takes part in the unfolded protein response (UPR) in the endoplasmic reticulum and is one of the signaling pathways that activate ERS. Under ERS, the expression of IRE1α is upregulated, and the activated IRE1α dissociates from GRP78 and undergoes phosphorylation. Activated IRE1’s activating enzyme activity further cleaves the mRNA of XBP to produce transcription factors encoding XBP1. It can act on the mRNA of XBP-1, splicing and removing 26 introns of XBP-1 mRNA, rapidly translating it into a large number of XBP1s, inducing the expression of molecular chaperones and folding enzymes, and promoting correct protein folding [35]. Under ERS, it causes an accumulation of a large number of unfolded proteins in the cell. These abnormally accumulated proteins activate the UPR through the IRE1/XBP1 pathway and induce cell apoptosis [14]. IRE1 α contains protein kinase and RNAse activity, activates the XBP1 protein, and participates in regulating the expression of downstream CHOP and other genes.

The activation of the IRE1α/XBP1 pathway is associated with apoptosis [36]. IRE1α overexpression can significantly downregulate the level of Polo-like kinase 1, thereby exacerbating the apoptosis level of liver cancer cells. In zebrafish models, the drug-induced activation of the ERS IRE1α/XBP1 pathway can promote lipid overproduction, which in turn affects glucose metabolism by regulating insulin levels, leading to liver cell apoptosis [37]. Additionally, upregulating the IRE1α/XBP1 pathway can further induce cell apoptosis by activating the JNK pathway [38]. Our results showed a significant increase in the GRP78 of mRNA and protein levels in the kidneys of Cd treatment group, indicating that Cd activates ERS, and the transmembrane protein dissociates from its molecular partner GRP78, resulting in an increase in GRP78 levels. At the same time, the mRNA and protein expression of GRP78 significantly decreased in the Cd + Que treatment group, indicating that Que has the ability to inhibit the dissociation of GRP78 to alleviate ERS. The gene and protein levels of IRE1 α and XBP1 significantly increased in the Cd treatment group. This discovery suggested that the elevated levels of GRP78 induced by cadmium treatment further activate the IRE1α-XBP1 signaling pathway of ERS. However, compared with those parameters in the Cd treatment group, the mRNA and protein expression contents of IRE1 α and XBP1 were reduced in the Cd + Que treatment group, indicating that Que can inhibit the expression of IRE1α signaling pathway-related genes in ERS, and to some extent alleviate the ERS caused by Cd. The function of ERS has a bidirectional regulatory effect, and the survival and death of cells are determined by the severity of ERS. Mild ERS induces an unfolded protein response, and promotes the correct folding of proteins, thereby alleviating ERS and exerting cell protective effects. However, long-term or severe ERS can trigger the apoptotic pathway, inducing cell apoptosis.

## 5. Conclusions

Cd activated the IRE1α-XBP1 signaling pathway of ERS, leading to renal cell apoptosis in rats. Que was found to protect against Cd-induced damage in kidney tissue and apoptosis through the IRE1α-XBP1 signaling pathway.

## Figures and Tables

**Table 1:** Primer sequences for the target genes.

Genes	Product Length	Primer Sequences (5′ → 3′)
*Caspase-12*	121 bp	Forward: TCGGAGAAGGAGCGAGCTTA Reverse: AGCTGTTTGTCGGAATTGGC
*Caspase-3*	156 bp	Forward: GCAGCAGCCTCAAATTGTTGACTA Reverse: TGCTCCGGCTCAAACCATC
*Bcl-2*	108 bp	Forward: CAAGCCGGGAGAACAGGGTA Reverse: CCCACCGAACTCAAAGAAGGC
*GRP78*	124 bp	Forward: ATGGTGTGGGAGATCCTGTTTTC Reverse: CAAGACGCACAGGGATACGC
*IRE1α*	98 bp	Forward: GCGCAGGTGCAATGACATAC Reverse: CATGCAAACTTCCGTCCAGG
*XBP1*	188 bp	Forward: CTGAGTCCGCAGCAGGTG Reverse: GACCTCTGGGAGTTCCTCCA
*β* *-actin*	168 bp	Forward: AGGGAAATCGTGCGTGACAT Reverse: CCTCGGGGCATCGGAA

## Data Availability

The raw data can be made available by the corresponding author upon request.
